# Two-hybrid screening of FAM13A protein partners in lung epithelial cells

**DOI:** 10.1186/s13104-019-4840-9

**Published:** 2020-01-03

**Authors:** Manon Ruffin, Kristin E. Thompson, Harriet Corvol, Loic Guillot

**Affiliations:** 1 Sorbonne Université, INSERM, Centre de Recherche Saint-Antoine, CRSA, Paris, France; 2 Pneumologie Pédiatrique, APHP, Hôpital Trousseau, Paris, France

**Keywords:** FAM13A, Chronic lung diseases, Lung epithelium, Two-hybrid screening

## Abstract

**Objectives:**

Family with sequence similarity 13 member A (FAM13A) genetic variants have been associated with several chronic respiratory diseases including chronic obstructive pulmonary disease (COPD), cystic fibrosis (CF), idiopathic pulmonary fibrosis (IPF) and lung cancer. The FAM13A protein includes a RhoGTPase activating protein (RhoGAP) domain known to participate in various cellular mechanisms including cell proliferation. While intensive genomic studies have been performed to reveal its involvement in lung diseases, the biological role of FAM13A protein is still not completely elucidated.

**Results:**

We therefore performed a two-hybrid screening to identify protein partners of FAM13A using a human lung cancer cDNA library. We identified several protein partners with a high confidence score. Researchers in the field of chronic lung diseases may benefit from this two-hybrid screening data which may reveal new research pathways to decipher.

## Introduction

*Family with sequence similarity 13 member A* (*FAM13A*) gene encodes for two proteins, isoform 1 and isoform 2. Isoform 1 contains a RhoGTPase activating protein (RhoGAP) domain known to play a role in cell cycle and proliferation [1]. Isoform 2 does not include this domain, suggesting that it has a function distinct from isoform 1. Initially, FAM13A was genetically associated with the lung function phenotype [2], with *FAM13A* genetic variants shown to be associated with lung cancer [3, 4] and several chronic lung diseases including chronic obstructive pulmonary disease (COPD) [5], cystic fibrosis (CF) [6], and idiopathic pulmonary fibrosis (IPF) [7]. However, despite this overlap of *FAM13A* contribution to chronic lung diseases, the several FAM13A variants have various impacts. Indeed, whether they are associated with an increase or decrease of the expression of the protein, their physiological consequences might be opposite [8]. Understanding the cellular role of FAM13A in the specific context of each of these diseases is thus essential.

The role of FAM13A in chronic lung diseases starts to be elucidated in various studies. A two-hybrid strategy aiming to identify partners of murine B56 family of phosphatase 2A (PP2A) regulatory subunits, identified FAM13A as a partner [9]. In COPD, by using affinity purification followed by mass spectrometry in HEK293 cells, the FAM13A isoform 2 has been shown to interact with PP2A and to be involved in the WNT/β-catenin pathway [10]. In CF, FAM13A is downregulated by Interleukin (IL)-1β and Transforming Growth Factor (TGF)-β, and is involved in the regulation of actin cytoskeleton dynamics and epithelial-mesenchymal transition [6]. In non-small lung cell cancer, FAM13A was shown to be involved in tumor proliferation downstream of HIF (Hypoxia Inducible Factor)-1α and TGF-β [11]. Besides, the involvement of FAM13A in IPF is still unknown. Outside the context of lung diseases, at the cellular level, FAM13A is able to control the cell shape [12].

The aim of this study was to identify the protein partners of the isoform 1 of FAM13A protein in order to decipher the pathways that may be affected in the different chronic lung diseases.

## Main text

### Methods

#### Yeast two-hybrid analysis

Yeast two-hybrid screening was performed by Hybrigenics Services, S.A.S., Paris, France (http://www.hybrigenics-services.com). The coding sequence for Human FAM13A full length (NCBI reference NM_014883.2) was from Origene (RC216561, Rockville, MD, USA) and cloned into pB27 as a C-terminal fusion to LexA (LexA-FAM13A). The construct was verified by sequencing the full insert and used as a bait to screen a random-primed Human Lung Cancer cDNA library constructed into pP6, pB27 and pP6 derivatives from the original pBTM116 [13] and pGADGH [14] plasmids, respectively. Also, the expected size of the FAM13A protein and RhoGAP activity was previously verified [6]. The Human Lung Cancer cDNA library is an equimolar mix of three different lung cancer cell lines: A549 (Human lung adenocarcinoma epithelial cell line), H1703 (Human squamous lung cancer cell line, adenocarcinoma; non-small cell lung cancer), H460 (lung carcinoma; large cell lung cancer, epithelial).

#### Technical validation

Screening of 64 million clones (sixfold the complexity of the library) was done using a mating method with YHGX13 (Y187 ade2-101:loxP-kanMX-loxP, matα) and L40∆Gal4 (mata) yeast strains as previously detailed [15]. 178 His+ colonies were selected on a medium without tryptophan, leucine and histidine, without 3-aminotriazole. The prey fragments of the positive clones were amplified by PCR and sequenced at their 5′ and 3′ junctions. Sequences were then used to identify the corresponding interacting proteins in the GenBank database [National Center for Biotechnology Information (NCBI)] using a fully automated procedure. A confidence score (PBS, for predicted biological score) was attributed to each interaction as previously described [16].

The PBS relies on two different levels of analysis. Firstly, a local score reflects the redundancy and independency of prey fragments, as well as the distribution of reading frames and stop codons in overlapping fragments. Secondly, a global score considers the interactions found in all the screens performed at Hybrigenics (proprietary database) using the same library. This global score represents the probability of an interaction to be nonspecific. The scores were divided into four categories, from A (highest confidence) to D (lowest confidence). A fifth category (E) particularly flags interactions involving highly connected prey domains previously discovered several times in screens accomplished on libraries derived from the same organism. Lastly, F corresponds to numerous of these highly connected domains confirmed as false-positives and are tagged F. The PBS scores have been shown to positively correlate with the biological significance of the interactions [17, 18].

#### Pathway analysis

Analysis of pathway ontology was realized with freely available PANTHER14.1 Released 2019-03-12 (Protein ANalysis THrough Evolutionary Relationships, http://pantherdb.org) [19]. Statistical enrichment pathway analysis was realized using as options: *Homo sapiens* reference list, PANTHER pathways dataset and Fisher’s Exact test followed by the calculation of false discovery rate (FDR).

### Results and discussion

We identified 17 proteins interacting with the FAM13A isoform 1, including some already shown to be involved in chronic lung diseases (Table 1 and Additional file 1).

**Table 1:** List of FAM13A interacting proteins detected by two-hybrid-screening

Protein	Gene	PBS	Association with lung disease	Ref.
Cilia- and flagella-associated protein 97	*CFAP97*	A	No	
Heat shock cognate 71 kDa protein	*HSPA8*	A	Involved in CFTR biogenesis and trafficking	[20]
			Regulated by corticoid in cell lysate of sputum of COPD patients	[21]
Serine/threonine-protein phosphatase 2A 56 kDa regulatory subunit epsilon isoform*	PPP2R5E*	A	Genetically associated with lung cancer	[22]
			PP2A activity was strongly enhanced in NSCLC	[23]
TBC1 domain family member 5	*TBC1D5*	A	Induced by smoking and ozone (murine COPD model)	[24]
Filamin-B	*FLNB*	C	No	
14-3-3 protein beta/alpha	*YWHAB*	C	Interact with and regulate surfactant protein A2	[25]
			Involved in lung cancer	[26]
Enhancer of mRNA-decapping protein 4	*EDC4*	C	No	
Tyrosine-protein phosphatase non-receptor type 12	*PTPN12*	D	High expression of PTPN12 is associated with favorable survival duration in patients with NSCLC	[27]
Histone-lysine N-methyltransferase	*SETMAR*	D	No	
tRNA cytosine [34]-C(5)-methyltransferase	*NSUN2*	D	No	
Retinal dehydrogenase 1	*ALDH1A1*	D	ALDH1 is a lung tumor stem cell-associated marker	[28]
			ALDH1 expression favorable prognosis in lung adenocarcinoma	[29]
Polyubiquitin-B	*UBB*	D	Reduced protein expression in COPD lung tissues	[30]
Eukaryotic initiation factor 4A-I	*EIF4A1*	D	No	
Serine/threonine-protein phosphatase 2A 56 kDa regulatory subunit alpha isoform**	PPP2R5A**	E	No	
14-3-3 protein epsilon	*YWHAE*	E	Upregulated in lung squamous cell carcinoma	[31]
14-3-3 protein zeta/delta	*YWHAZ*	E	Identified as a metastasis enhancer gene in lung cancer	[32]
			Upregulated in lung cancer	[33]
			Differentially expressed in malignant bronchial epithelial cell line compared to control cell line	[34]
			More frequent increased expression in patients with resectable lung adenocarcinoma with an improved prognosis	[35]
26S proteasome non-ATPase regulatory subunit 11	*PSMD11*	F	Differentially expressed in malignant bronchial epithelial cell line compared to control cell line	[34]

*PBS* Predicted Biological Score [9]; *NSCLC* non-small cell lung cancer

*Interaction previously shown using an adult mouse brain library and PPP2R5E (full-length *Xenopus* B56ε) or **mouse FAM13A as bait in two-hybrid screening

**Fig. 1:**
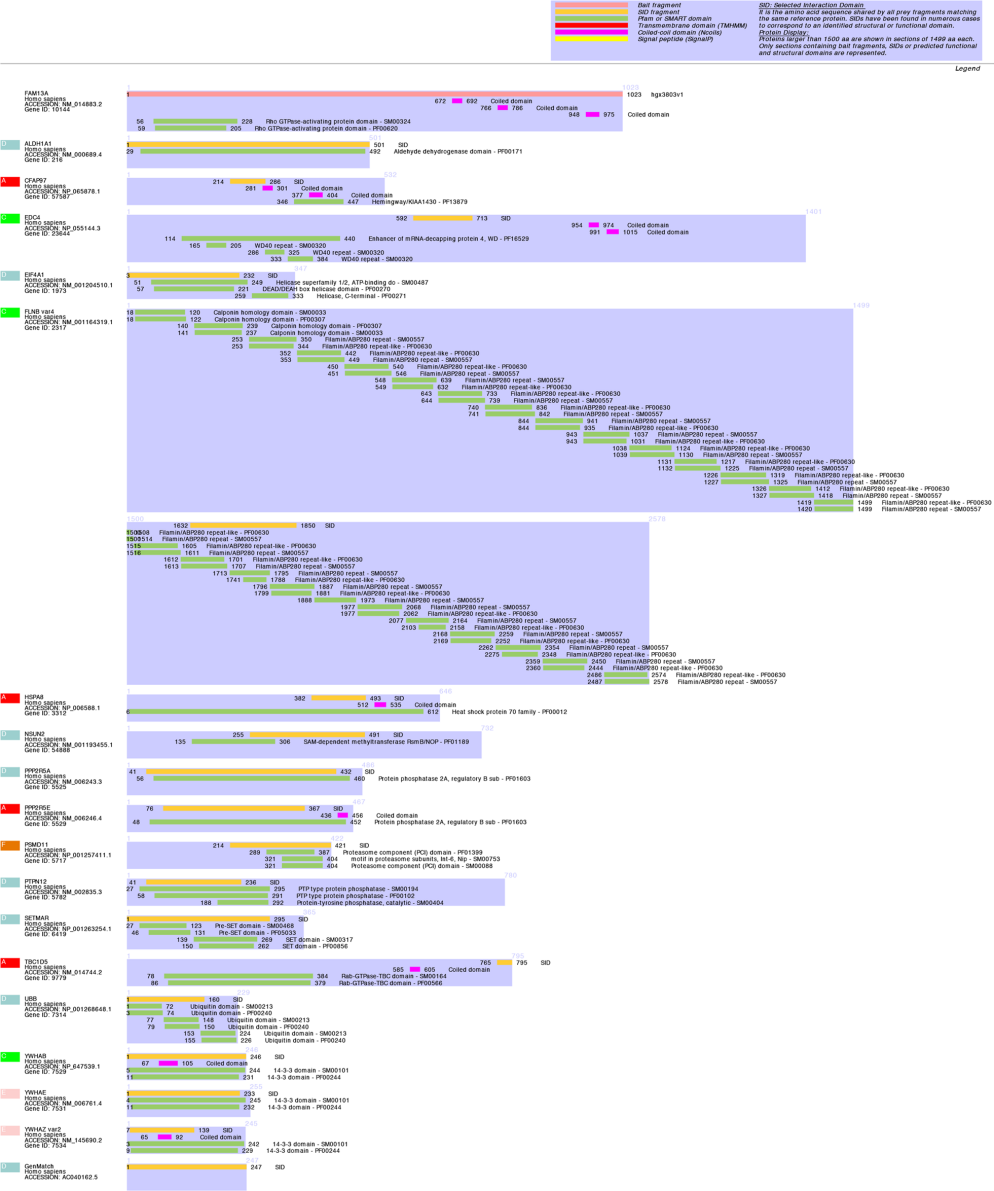
Diagram of the proteins identified in the two-hybrid screening and their interaction domains

Interaction domains are depicted in Fig. 1. Four proteins were detected with a high predicted Biological Score (PBS): CFAP97, HSPA8, PPP2R5E and TBC1D5. HSPA8 is well known to be involved in Cystic Fibrosis Transmembrane conductance Regulator (CFTR) biogenesis and trafficking [20]. *CFTR* is the causative gene of CF. This FAM13A-HSPA8 interaction may be important in the role of FAM13A as a modifier of the CF lung phenotype as previously described [6]. Interestingly, we detected two isoforms of the protein phosphatase 2A (PP2A) B subunit, PPP2R5E and PPP2R5A, previously shown to interact with FAM13A [9, 10], thus verifying the success of our two-hybrid screening. The PP2A protein has been associated with several lung diseases. In CF, PP2A is known to regulate CFTR activity [36]. In COPD, FAM13A was shown to recruit PP2A and influence disease susceptibility by promoting β-catenin degradation [10]. Recent works highlight PP2A as a promising therapeutic target for chronic lung diseases. Indeed, enhancement of PP2A activity was recently shown to reduce cigarette smoke-induced cathepsin S and loss of lung function [37] and to improve the treatment of tyrosine kinase inhibitor-resistant lung adenocarcinoma [38]. How FAM13A-PP2A interaction could be involved in these processes remains to be elucidated.

**Fig. 2:**
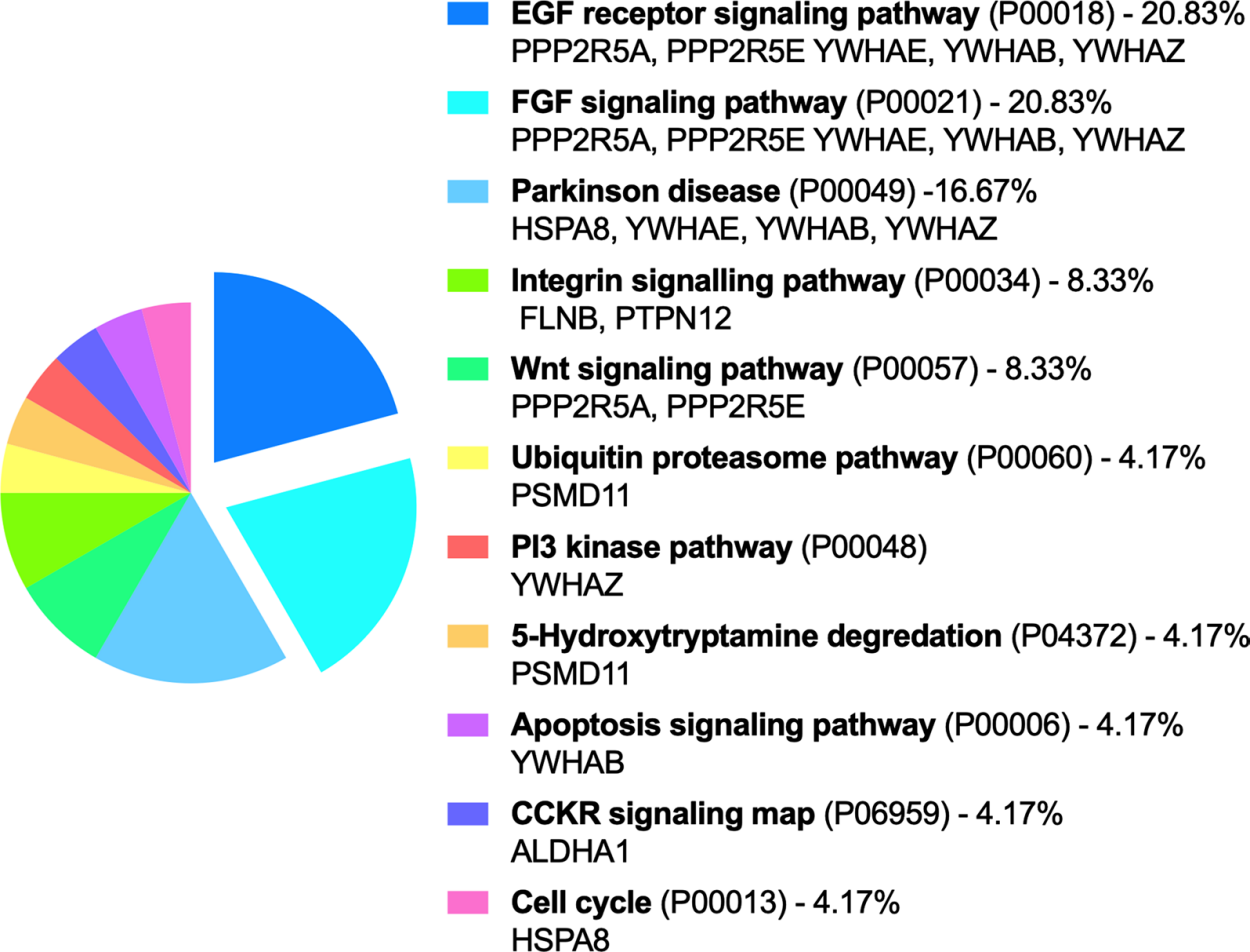
Pathway ontology analysis. Analysis of pathway ontology was realized with PANTHER14.1 Released 2019-03-12 (Protein ANalysis THrough Evolutionary Relationships, http://pantherdb.org) [19]. PANTHER Pathways references are given as well as the percent of gene hits against total genes

Pathway enrichment statistical analysis found three major pathways: Fetal Growth Factor (FGF) signaling pathway (FDR = 6.59 × 10^−6^), EGF receptor (EGFR) signaling pathway (FDR = 5.61 × 10^−6^) and Parkinson disease (FDR = 7.01 × 10^−5^) (Fig. 2). In FGF and EGFR signaling pathways, PP2A B subunit isoforms (PPP2R5A, PPP2R5E) and 14-3-3 proteins (YWHAB, YWHAZ, YWHAE) were shared. 14-3-3 proteins are especially involved in lung cancer [39]. Interestingly, YWHAB was also shown to interact with surfactant protein A2 (SP-A2) [25] and genetic variants of YWHAB can predispose individuals to IPF and lung cancer [40]. Since FAM13A was also genetically associated with IPF and lung cancer, further studies may help to understand the consequences of these interactions in the development of these diseases.

In conclusion, we confirmed and identified new protein partners of FAM13A. The future study of these interactions may help to not only understand the overlapping role of FAM13A in chronic lung diseases but their etiology as well.

## Limitations

The limitation of the study is the use of a Human Lung Cancer cDNA library as a prey. Indeed, the proteins identified to interact with FAM13A may be specific of the cancer origins of the cells and my different in a non-pathological context. Also, additional methods will be necessary to confirm these interactions.

## Supplementary information

**Additional file 1.** Two-hybrid sequence data.

## Data Availability

The protein interactions data from this publication have been submitted to the IMEx (http://www.imexconsortium.org) consortium through IntAct [41] and assigned the identifier IM-27362 (Link: www.ebi.ac.uk/intact/search/do/search?searchString=pubid:IM-27362). Confidence score (PBS, for predicted biological score) calculations are from a not available proprietary database (Hybrigenics).

## Abbreviations

FAM13A: family with sequence similarity 13 member A
COPD: chronic obstructive pulmonary disease
CF: cystic fibrosis
IPF: idiopathic pulmonary fibrosis
RhoGAP: RhoGTPase activating protein
(IL)-1β: Interleukin
(TGF)-β: Transforming Growth Factor
(HIF)-1α: Hypoxia Inducible Factor
PBS: predicted biological score
NCBI: National Center for Biotechnology Information
PANTHER: Protein ANalysis THrough Evolutionary Relationships
FDR: false discovery rate
CFTR: Cystic Fibrosis Transmembrane conductance Regulator
PP2A: protein phosphatase 2A
FGF: Fetal Growth Factor
EGFR: EGF receptor
SP-A2: surfactant protein A2

## References

[1] Corvol H, Hodges CA, Drumm ML, Guillot L (2014). Moving beyond genetics: is FAM13A a major biological contributor in lung physiology and chronic lung diseases?. J Med Genet.

[2] Hancock DB, Eijgelsheim M, Wilk JB, Gharib SA, Loehr LR, Marciante KD (2010). Meta-analyses of genome-wide association studies identify multiple loci associated with pulmonary function. Nat Genet.

[3] Ziolkowska-Suchanek I, Mosor M, Gabryel P, Grabicki M, Zurawek M, Fichna M (2015). Susceptibility loci in lung cancer and COPD: association of IREB2 and FAM13A with pulmonary diseases. Sci Rep..

[4] Young RP, Hopkins RJ, Hay BA, Whittington CF, Epton MJ, Gamble GD (2011). FAM13A locus in COPD is independently associated with lung cancer—evidence of a molecular genetic link between COPD and lung cancer. Appl Clin Genet..

[5] Cho MH, Boutaoui N, Klanderman BJ, Sylvia JS, Ziniti JP, Hersh CP (2010). Variants in FAM13A are associated with chronic obstructive pulmonary disease. Nat Genet.

[6] Corvol H, Rousselet N, Thompson KE, Berdah L, Cottin G, Foussigniere T (2018). FAM13A is a modifier gene of cystic fibrosis lung phenotype regulating rhoa activity, actin cytoskeleton dynamics and epithelial-mesenchymal transition. J Cyst Fibros.

[7] Hobbs BD, de Jong K, Lamontagne M, Bosse Y, Shrine N, Artigas MS (2017). Genetic loci associated with chronic obstructive pulmonary disease overlap with loci for lung function and pulmonary fibrosis. Nat Genet.

[8] van Moorsel CHM (2018). Trade-offs in aging lung diseases: a review on shared but opposite genetic risk variants in idiopathic pulmonary fibrosis, lung cancer and chronic obstructive pulmonary disease. Curr Opin Pulm Med..

[9] Jin Z, Chung JW, Mei W, Strack S, He C, Lau GW (2015). Regulation of nuclear-cytoplasmic shuttling and function of Family with sequence similarity 13, member A (Fam13a), by B56-containing PP2As and Akt. Mol Biol Cell.

[10] Jiang Z, Lao T, Qiu W, Polverino F, Gupta K, Guo F (2016). A chronic obstructive pulmonary disease susceptibility gene, FAM13A, regulates protein stability of beta-catenin. Am J Respir Crit Care Med.

[11] Eisenhut F, Heim L, Trump S, Mittler S, Sopel N, Andreev K (2017). FAM13A is associated with non-small cell lung cancer (NSCLC) progression and controls tumor cell proliferation and survival. Oncoimmunology.

[12] Pascual-Vargas P, Cooper S, Sero J, Bousgouni V, Arias-Garcia M, Bakal C (2017). RNAi screens for Rho GTPase regulators of cell shape and YAP/TAZ localisation in triple negative breast cancer. Sci Data..

[13] Vojtek AB, Hollenberg SM (1995). Ras-Raf interaction: two-hybrid analysis. Methods Enzymol.

[14] Bartel PL, Fields S (1995). Analyzing protein-protein interactions using two-hybrid system. Methods Enzymol.

[15] Fromont-Racine M, Rain JC, Legrain P (1997). Toward a functional analysis of the yeast genome through exhaustive two-hybrid screens. Nat Genet.

[16] Formstecher E, Aresta S, Collura V, Hamburger A, Meil A, Trehin A (2005). Protein interaction mapping: a Drosophila case study. Genome Res.

[17] Wojcik J, Boneca IG, Legrain P (2002). Prediction, assessment and validation of protein interaction maps in bacteria. J Mol Biol.

[18] Rain JC, Selig L, De Reuse H, Battaglia V, Reverdy C, Simon S (2001). The protein-protein interaction map of *Helicobacter pylori*. Nature.

[19] Mi H, Muruganujan A, Huang X, Ebert D, Mills C, Guo X (2019). Protocol update for large-scale genome and gene function analysis with the PANTHER classification system (v.14.0). Nat Protoc.

[20] Meacham GC, Lu Z, King S, Sorscher E, Tousson A, Cyr DM (1999). The Hdj-2/Hsc70 chaperone pair facilitates early steps in CFTR biogenesis. EMBO J.

[21] Holownia A, Mroz RM, Kielek A, Chyczewska E, Braszko JJ (2009). Nuclear HSP90 and HSP70 in COPD patients treated with formoterol or formoterol and corticosteroids. Eur J Med Res..

[22] Baumgartner U, Berger F, Hashemi Gheinani A, Burgener SS, Monastyrskaya K, Vassella E (2018). miR-19b enhances proliferation and apoptosis resistance via the EGFR signaling pathway by targeting PP2A and BIM in non-small cell lung cancer. Mol Cancer..

[23] Yang R, Yang L, Qiu F, Zhang L, Wang H, Yang X (2013). Functional genetic polymorphisms in PP2A subunit genes confer increased risks of lung cancer in southern and eastern Chinese. PLoS ONE.

[24] Uh ST, Koo SM, Jang AS, Park SW, Choi JS, Kim YH (2015). Proteomic differences with and without ozone-exposure in a smoking-induced emphysema lung model. Korean J Intern Med.

[25] Noutsios GT, Silveyra P, Bhatti F, Floros J (2013). Exon B of human surfactant protein A2 mRNA, alone or within its surrounding sequences, interacts with 14-3-3; role of cis-elements and secondary structure. Am J Physiol Lung Cell Mol Physiol.

[26] Okayama A, Miyagi Y, Oshita F, Nishi M, Nakamura Y, Nagashima Y (2014). Proteomic analysis of proteins related to prognosis of lung adenocarcinoma. J Proteome Res.

[27] Cao X, Chen YZ, Luo RZ, Zhang L, Zhang SL, Zeng J (2015). Tyrosine-protein phosphatase non-receptor type 12 expression is a good prognostic factor in resectable non-small cell lung cancer. Oncotarget..

[28] Jiang F, Qiu Q, Khanna A, Todd NW, Deepak J, Xing L (2009). Aldehyde dehydrogenase 1 is a tumor stem cell-associated marker in lung cancer. Mol Cancer Res.

[29] Koh YW, Han JH, Haam S, Jung J (2019). ALDH1 expression correlates with an epithelial-like phenotype and favorable prognosis in lung adenocarcinoma: a study based on immunohistochemistry and mRNA expression data. J Cancer Res Clin Oncol.

[30] Stepaniants S, Wang IM, Boie Y, Mortimer J, Kennedy B, Elliott M (2014). Genes related to emphysema are enriched for ubiquitination pathways. BMC Pulm Med..

[31] Gan XN, Gan TQ, He RQ, Luo J, Tang RX, Wang HL (2018). Clinical significance of high expression of miR-452-5p in lung squamous cell carcinoma. Oncol Lett..

[32] Chen JJ, Peck K, Hong TM, Yang SC, Sher YP, Shih JY (2001). Global analysis of gene expression in invasion by a lung cancer model. Cancer Res.

[33] Fan T, Li R, Todd NW, Qiu Q, Fang HB, Wang H (2007). Up-regulation of 14-3-3zeta in lung cancer and its implication as prognostic and therapeutic target. Cancer Res.

[34] Liu XD, Xie DF, Wang YL, Guan H, Huang RX, Zhou PK (2019). Integrated analysis of lncRNA-mRNA co-expression networks in the alpha-particle induced carcinogenesis of human branchial epithelial cells. Int J Radiat Biol.

[35] Li M, Lu H, Liu X, Meng Q, Zhao Y, Chen X (2018). Overexpression of 14-3-3zeta in lung tissue predicts an improved outcome in patients with lung adenocarcinoma. Oncol Lett..

[36] Berger HA, Travis SM, Welsh MJ (1993). Regulation of the cystic fibrosis transmembrane conductance regulator Cl-channel by specific protein kinases and protein phosphatases. J Biol Chem.

[37] Doherty DF, Nath S, Poon J, Foronjy RF, Ohlmeyer M, Dabo AJ (2019). Protein phosphatase 2A reduces cigarette smoke-induced cathepsin S and Loss of Lung Function. Am J Respir Crit Care Med.

[38] Tohme R, Izadmehr S, Gandhe S, Tabaro G, Vallabhaneni S, Thomas A (2019). Direct activation of PP2A for the treatment of tyrosine kinase inhibitor-resistant lung adenocarcinoma. JCI Insight..

[39] Khorrami A, Sharif Bagheri M, Tavallaei M, Gharechahi J (2017). The functional significance of 14-3-3 proteins in cancer: focus on lung cancer. Horm Mol Biol Clin Investig..

[40] Wang Y, Kuan PJ, Xing C, Cronkhite JT, Torres F, Rosenblatt RL (2009). Genetic defects in surfactant protein A2 are associated with pulmonary fibrosis and lung cancer. Am J Hum Genet.

[41] Orchard S, Ammari M, Aranda B, Breuza L, Briganti L, Broackes-Carter F (2014). The MIntAct project–IntAct as a common curation platform for 11 molecular interaction databases. Nucleic Acids Res.

